# Runs of Homozygosity Associated with Speech Delay in Autism in a Taiwanese Han Population: Evidence for the Recessive Model

**DOI:** 10.1371/journal.pone.0072056

**Published:** 2013-08-16

**Authors:** Ping-I Lin, Po-Hsiu Kuo, Chia-Hsiang Chen, Jer-Yuarn Wu, Susan S-F. Gau, Yu-Yu Wu, Shih-Kai Liu

**Affiliations:** 1 Division of Biostatistics and Epidemiology, Cincinnati Children’s Hospital Medical Center, Cincinnati, Ohio, United States of America; 2 Department of Psychiatry, National Taiwan University Hospital, Taipei, Taiwan; 3 Graduate Institute of Epidemiology and Preventive Medicine, National Taiwan University College of Public Health, Taipei, Taiwan; 4 Department of Psychiatry, National Taiwan University College of Medicine, Taipei, Taiwan; 5 Center for Neuropsychiatric Research, National Health Research Institutes, Zhunan, Taiwan; 6 Institute of Biomedical Sciences, Academia Sinica, Taipei, Taiwan; 7 School of Chinese Medicine, China Medical University, Taichung, Taiwan; 8 Graduate Institute of Brain and Mind Sciences, Graduate Institute of Clinical Medicine, Department of Psychology, and School of Occupational Therapy, National Taiwan University, Taipei, Taiwan; 9 Department of Psychiatry, Chang Gung Memorial Hospital- Linkou Medical Center, Chang Gung University College of Medicine, Tao-Yuan, Taiwan; 10 Department of Child and Adolescent Psychiatry, Taoyuan Mental Hospital, Department of Health, Executive Yuan, Tao-Yuan, Taiwan; Sanjay Gandhi Medical Institute, India

## Abstract

Runs of homozygosity (ROH) may play a role in complex diseases. In the current study, we aimed to test if ROHs are linked to the risk of autism and related language impairment. We analyzed 546,080 SNPs in 315 Han Chinese affected with autism and 1,115 controls. ROH was defined as an extended homozygous haplotype spanning at least 500 kb. Relative extended haplotype homozygosity (REHH) for the trait-associated ROH region was calculated to search for the signature of selection sweeps. Totally, we identified 676 ROH regions. An ROH region on 11q22.3 was significantly associated with speech delay (corrected *p* = 1.73×10^−8^). This region contains the NPAT and ATM genes associated with ataxia telangiectasia characterized by language impairment; the CUL5 (culin 5) gene in the same region may modulate the neuronal migration process related to language functions. These three genes are highly expressed in the cerebellum. No evidence for recent positive selection was detected on the core haplotypes in this region. The same ROH region was also nominally significantly associated with speech delay in another independent sample (*p* = 0.037; combinatorial analysis Stouffer’s *z* trend = 0.0005). Taken together, our findings suggest that extended recessive loci on 11q22.3 may play a role in language impairment in autism. More research is warranted to investigate if these genes influence speech pathology by perturbing cerebellar functions.

## Introduction

Autistic disorder (henceforth denoted as autism) is a neurodevelopmental disorder characterized by deficits in communication, social interaction, and behavioral patterns. Family and twin studies have strongly suggested that genetic factors contribute to the development of autism [1]. Most genome-wide association studies (GWAS) have investigated the impact of genetic variants on the risk of autism one at a time [2]–[4]. However, many of these GWAS-derived findings could not be successfully replicated across different populations [5]. The failure to replicate previous findings may be, at least in part, attributed to the negligence of multi-locus effects [6]. To evaluate all possible multi-locus effects in the context of hypothesis-free GWAS, one has to overcome the computational and statistical burden. Some of prior studies have focused on genes with relevant biological functions to investigate multi-locus effects on the risk of autism [7]–[9]. Additionally, whole-genome scans also suggest that a cluster of rare variants across different genes may collectively predict the risk of autism [10], [11]. Therefore, systemic approaches to investigating the effect of clusters of multiple loci from the whole genome may lead to discoveries that complement the GWAS-derived findings.

Runs of homozygosity (ROHs) may play a role in neuropsychiatric diseases, such as schizophrenia [12], [13] and Alzheimer’s disease [14]. A recent study also identified several novel candidate genes characterized by ROHs associated with the risk of autism [15]. Compared to the number of SNPs in the whole genome, the number of ROHs is apparently more tractable, and hence requires a less stringent significance threshold to search for significant findings. Therefore, a ROH-based approach may provide opportunities of revealing multi-locus effects on phenotypes. The link between common ROHs and diseases may reflect several different non-mutually exclusive mechanisms. First, a haplotype at high frequency with high homozygosity spanning over a large region is a sign of an incomplete selective sweep. Under such circumstances, an individual may carry consecutive homozygous SNPs due to identical-by-descent haplotypes that harbor ancestral alleles with an advantageous effect [16]. In case-control studies, an ROH over-represented in cases may be attributed to a disease-linked variant with an advantageous effect, while an ROH over-represented in controls may stem from a protective effect of recent mutation. On the other hand, selection pressure may not purge all deleterious mutations and hence inbreeding might cause the accumulation of multiple variants of adverse effects, which leads to a multi-locus recessive disease model. Alternatively, a disease-associated ROH may arise when a deleterious mutation is in linkage disequilibrium with another variant that undergoes recent positive selection [17]. Second, an ROH over-represented in cases may simply stem from a multi-locus recessive disease model. Third, a disease-associated ROH may indicate the difference in relatedness between cases and controls [18]. The ROH-based analysis is a novel approach to identifying clustering patterns of variants to unmask ambiguous disease-genotype associations. To explore the relationships between ROHs and autism, we conducted a genome-wide association study in a Taiwanese Han population. Our core hypothesis posits that several novel genes characterized by ROHs are associated with autism and its related language impairment. We selected speech delay as the primary clinical feature as previous evidence suggests that language impairment is the most important predictor for the prognosis and developmental course of autism [19], [20].

## Results

**Figure 1:**
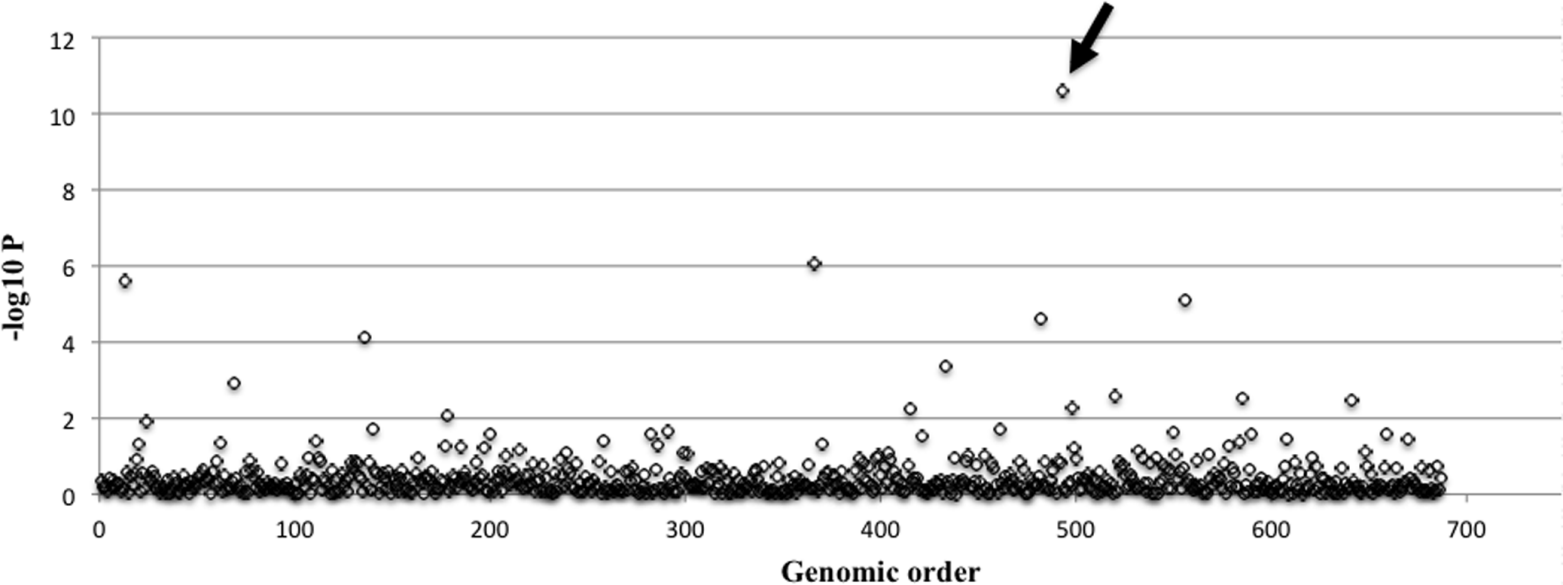
The association findings for age of first phrase (AFP) are presented as –log_10_ p-values (unadjusted by multiple tests) across the whole autosome. The arrow indicates the ROH region at 11q22.3.

**Figure 2:**
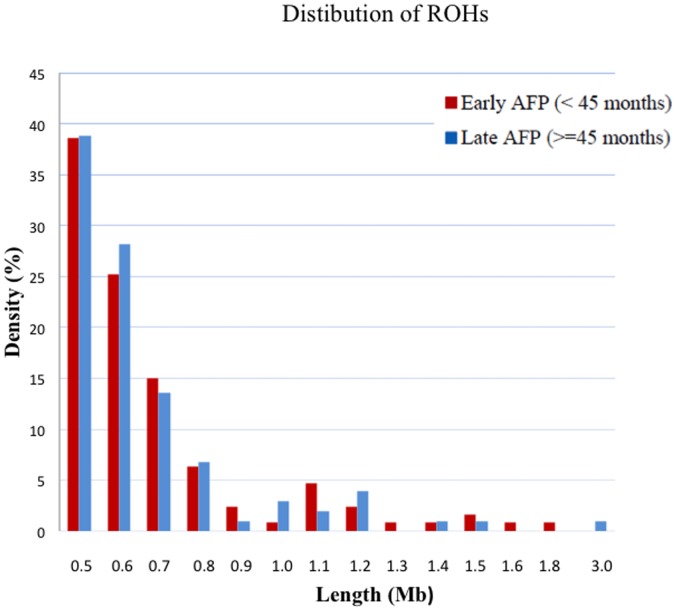
The distribution of runs of homozygosity (ROH) regions (by length of the ROH region) is shown. Age of first phrase (AFP) was classified into early-AFP and late-AFP groups by the k-means clustering algorithm.

The descriptive analysis results for demographic and clinical features are summarized in Table 1. Verbal IQ and Performance IQ had the highest percentage of missing data, and hence we compared the association test results with and without Verbal IQ/Performance IQ in the regression model. Since the effect of IQ on the association between ROH markers and traits was limited, the missing data of IQ might not pose a great concern in the current study. The case-control association analysis did not yield genome-wide significant findings after multiple-testing correction (Table 2). There was no statistically significant difference in the ROH length between cases and controls (mean length: 658 kb vs. 645 kb; z = 0.62, *p* = 0.229). We also calculated the value of Froh (total length of all their ROHs in the autosome and divided by the total SNP-mappable autosomal distance) for the ROH burden analysis [21], and did not find any significant difference in Froh between and cases and controls. The genome-wide significant finding (*p* = 0.05/676 = 7.4×10^−5^) was obtained from the association analysis for speech delay (surrogated by “age-of-first-phrase or AFP”). The association analysis findings across the whole autosome are illustrated in Figure 1. We also used k-means clustering algorithm to classify the population into two subgroups, and identified the early-AFP group and late-AFP group with the cutoff at 45 months of age. The results suggest that the distributions of ROHs of early-AFP versus late-AFP groups appeared to be similar to each other (Figure 2). One ROH region on chromosome 11q22.3 was significantly associated with risk of speech delay (Bonferroni-corrected *p* = 1.73×10^−8^). The significant results (corrected with Bonferroni method) for AFP are summarized in Table 3. This ROH marker was found to be positively associated with AFP as a continuous variable. This result remained statistically genome-wide significant after we adjusted for IQ, gender, and education levels of parents. We also assessed the relationship between this ROH region and AFP as a dichotomous variable using the logistic regression model, and the association remained significant (p<0.0001). This region contains nine genes, none of which has been found to be associated with the risk of autism in previous studies or specific language disorders.

**Table 1:** Demographic features of cases in the discovery population.

Variable	Minimum	Median	Mean	Maximum	Standard Deviation	# Missing	# Non-missing
Maternal Education^1^	1	4	3.7	5	0.76	0	315
Paternal Education^1^	1	4	3.9	5	0.77	0	315
SRST^2^	32	97.0	99.4	183	26.63	11	304
SCQ^3^	3	18	18.19	36	7.03	59	256
FSTBEH^4^	3	18	18.66	37	6.76	58	257
Age of First Phrase^5^	5	36.0	39.6	156	18.29	85	230
VIQ^6^	44	100.0	95.2	148	24.24	114	201
PIQ^6^	41	98.0	97.1	145	20.71	114	201
Paternal age^7^	22.8	33.7	34.1	59.3	4.88	57	258
Maternal age^7^	19.6	30.5	30.9	44.5	4.53	57	258

1 Maternal and paternal education: 1≤6 years, 2 = 7−9 years; 3 = 10−12 years, 4 = 13−16 years, 5 = >16 years.

2 SRST: Total social responsiveness score assessed by the Autism Diagnostic Interview-revised.

3 SCQ: social communication quotient total score.

4 FSTBEH: Stereotype behavior/interest score assessed by the Autism Diagnostic Interview-revised.

5 The unit of age of phrase is month.

6 VIQ = verbal IQ; PIQ = performance IQ.

7 The unit of paternal/maternal age is year.

**Table 2:** Case-control association test results for 4 runs of homozygosity (ROH) regions nominally associated with the risk of autism (unadjusted p-value <0.01) in the discovery sample.

Start position	End position	Length	Chromo-some	Fisher PΔ	Odds ratio	R_Case_ *	R_Ctrl_ *	Genes
208,824,998	209,180,938	355,940	2	0.000827	20.32	0.018	0.001	IDH1, PIP5K3, PTH2R
120,293,987	120,445,970	151,983	8	0.000827	20.32	0.018	0.001	MAL2
46,630,743	47,455,038	824,295	20	0.002221	5.10	0.027	0.005	PREX1, ARFGEF2, FKSG61, CSE1L, CSE1, STAU1, DDX27, ZNFX1, C20orf199, KCNB1
120,782,861	121,320,700	537,839	8	0.006451	6.76	0.018	0.003	TAF2, DSCC1, DEPDC6, COL14A1

* R_Case_ = prevalence rate of the ROH marker in cases; R_Ctrl_ = prevalence rate of the ROH marker in controls;

Δ Unadjusted p-values based on the Fisher’s exact tests.

**Table 3:** Case-only association test results for age of first phrase (only unadjusted p-value <1×10^−5^ were shown).

Start position	End position	Length	Chrom		Regression coefficient	P_Bonf_ *	Genes	
107,309,153	108,201,671	892,518	11		116.9(±10.6)	1.7×10^−8^	RAB39, CUL5, ACAT1, NPAT, ATM, C11orf65, KDELC2, EXPH, DOX10	
89,171,609	90,594,484	1,422,875	7		51.08(±6.1)	7.3×10^−7^	DPY19L2P4, STEAP1, STEAP2, C7orf63, GTPBP10, CLDN12, PFTK1, KIAA0834	
42,126,282	43,130,565	1,004,283	1		49.05(±5.6)	0.0035	GUCA2B, FOXJ3, ZMYND12, RIMKLA, PPCS, LOC728621, DKFZp686K01114, PPIH, AF086102, YBX1, CLDN19, LEPRE1, CR623026, C1orf50, CCDC23, ERMAP, ZNF691	
76404435	77605674	1,201,239		13	56.91(±5.1)	0.0113	CLN5, FBXL3, KIAA0916, MYCBP2, DKFZp586G0322, SEL, SLAIN1, EDNRB, AK095779	

* Bonferroni-corrected p-value.

**Figure 3:**
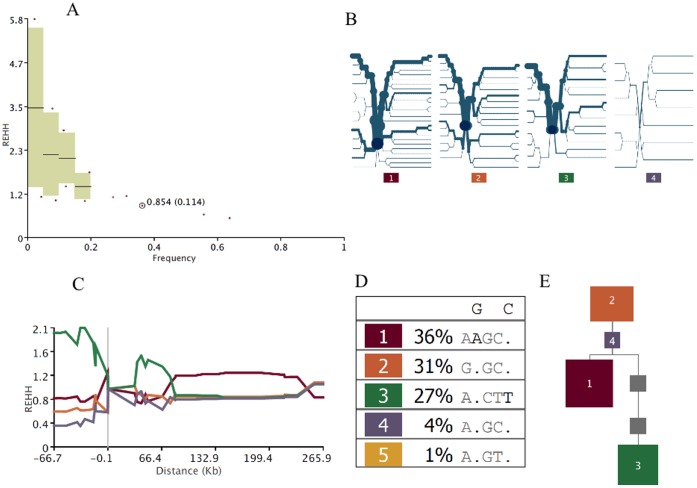
The analysis results based on the early-AFP group are shown. Panel A shows the scatter plot of REHH plotted against all core haplotype frequency (circled dot indicates the selected core haplotype “rs1074014-rs1072877-rs1564582-rs11212724-rs11211725”). Panel B shows the haplotype bifurcation diagram, which visualizes the breakdown of LD at increasing distances from core haplotypes at the selected core region. The root of each diagram is a core haplotype, identified by a dark blue circle. Panel C illustrates how the REHH value varies by the selected core haplotype. Panel D shows the table of core haplotype, and the dot in the observed haplotype sequence represents the allele that matches the ancestral. Panel E presents the theoretical phylogenetic tree of different core haplotypes. Gray squares represent haplotypes that are not present in the observed data, but are missing links in the phylogeny. The area of the squares is proportional to the frequency of the haplotype.

**Figure 4:**
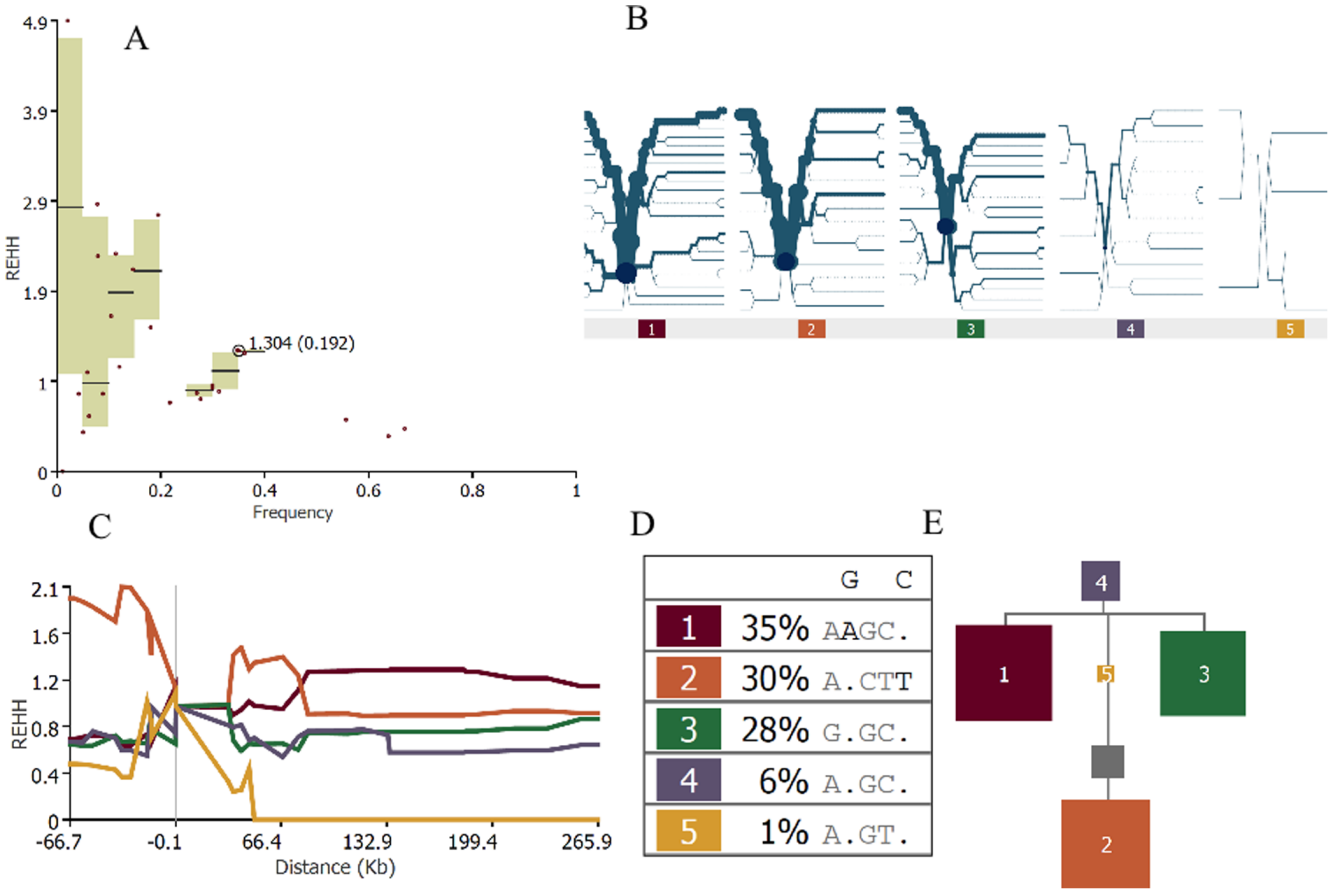
The analysis results based on the late age of first phrase (late-AFP) group are shown. Panel A shows the scatter plot of REHH plotted against all core haplotype frequency (circled dot indicates the selected core haplotype “rs1074014-rs1072877-rs1564582-rs11212724-rs11211725”). Panel B shows the haplotype bifurcation diagram, which visualizes the breakdown of LD at increasing distances from core haplotypes at the selected core region. The root of each diagram is a core haplotype, identified by a dark blue circle. Panel C illustrates how the REHH value varies by the selected core haplotype. Panel D shows the table of core haplotype, and the dot in the observed haplotype sequence represents the allele that matches the ancestral. Panel E presents the theoretical phylogenetic tree of different core haplotypes. Gray squares represent haplotypes that are not present in the observed data, but are missing links in the phylogeny. The area of the squares is proportional to the frequency of the haplotype.

**Figure 5:**
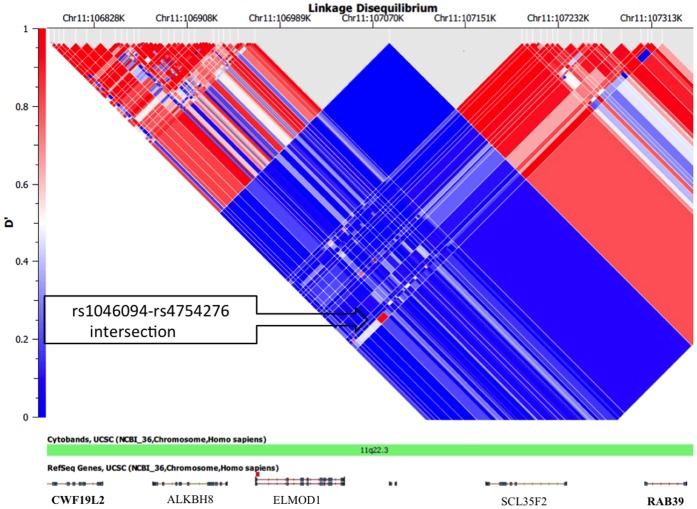
Linkage disequilibrium patterns in the 11q22.3 region are shown.

We further examined if the ROH region on 11q22.3 might arise from selection sweeps. The distributions of relative extended haplotype homozygosity (REHH) of the early-AFP and late-AFP groups (classified using the k-means clustering algorithm) appeared to be similar to each other (Figure 3A versus Figure 4A). The distributions of REHH (the factor by which EHH decays on the tested five-SNP core haplotype “rs1074014-rs1072877-rs1564582-rs11212724-rs11211725” in the genes on 11q22.3) of early-AFP and late-AFP groups seemed to differ by the core haplotype with strongest evidence for incomplete selection sweep (Figure 3C versus Figure 4C). Additionally, these two groups might have different ancestral haplotypes (Figure 3E versus 4E), although their frequency distributions of core haplotypes were similar (Figure 3B, 3D versus Figure 4B, 4D). The Nevertheless, none of these core haplotypes appeared to have remarkable signatures of recent positive selection based on the REHH distributions (i.e., REHH exceeding 2 at 200 Kb away from the core haplotype). We also searched for the signature of selection sweeps in genes proximal to 11q22.3, and found that the CWF19L2 (CWF19-like 2, cell cycle control) gene located 1 Mb upstream to this region based on the phase-I Hapmap Asian-descent population (CHB+JPT) has an iHS (Integrated Haplotype Score ) score = 1.7 (*p* = 0.0237) based on the query using the webtool Haplotter [22]. We hence calculated the linkage disequilibrium (LD) coefficients D′ between the ROH region and CWF19L2 gene, and found that a locus (rs1046094, a 3′ UTR variant) within the CWF19L2 gene was correlated with another locus (rs4754276, an intronic variant) within the RAB39 (ras-related protein Rab-39A) gene (D′ = 0.9) (Figure 5).

The inbreeding coefficient F value was <0.01 based on the SNP data on chromosome 11 in either the early-AFP or late-AFP groups. Therefore, the ROH markers associated with speech delay might not be caused by the difference in the degree of consanguinity between these two subpopulations. Additionally, we queried the CNV data generated by the same SNP arrays in the discovery sample, and did not find any deletions or duplications in this 11q region. Therefore, the ROHs on 11q22.3 were not likely attributed to hemizygous deletions.

### Replication Study

The recruitment of subjects under the auspice of Autism Genetic Resource Exchange (AGRE) has been described elsewhere [23]. Briefly, AGRE is a joint effort of the Cure Autism Now (CAN) Foundation and the Human Biological Data Interchange (HBDI). The diagnosis was made by all of the NIH autism collaborative groups using the Autism Diagnostic Interview–Revised (ADI-R) [24] and the Autism Diagnostic Observational Schedule (ADOS) [25]. We have downloaded the clinical and SNP data (generated by the Affymetrix SNP 5.0 platform) for all probands. We implemented the same data-cleaning algorithm used in the discovery sample. A total of 325,971 valid SNPs for 1,387 subjects diagnosed with autism were obtained. The age of first phrase (AFP) distributions of the AGRE sample and our discovery sample are shown in Supporting Information (Figure S1). We did not find significant difference in the distributions of AFP between the discovery population (Taiwan) and replication sample (AGRE) (Mann-Whitney U test p>0.05). We attempted to replicate the association between the ROH region on 11q22.3 and AFP in another independent population. The SNP data on chromosome 11 were retrieved from 1,387 individuals affected with autism recruited through multi-site collaborative efforts of Autism Genetic Resource Exchange (AGRE). We performed the same statistical methods as what we used in the discovery sample described in the Methods section and identified 31 ROH regions on chromosome 11. When AFP was treated as a continuous outcome, no significant association was detected on 11q22.3. However, when we chose 49 months as a cutoff using the k-means clustering algorithm to define the presence of “speech delay,” we found that the ROH region on 11q22.3 (117.5 Mb-113.1 Mb) was nominally significantly associated with speech delay (*P* = 0.0377). We then calculated the combined p-values from these two samples based on the Stouffer method, and obtained Stouffer z value and z trend of 0.0007 and 0.0005, respectively. Note that these SNP data were based on Affymetrix SNP 5.0 platform that had lower marker density than Affymetrix SNP 6.0 data. The AGRE sample had a European origin, which might also contribute to different ROH patterns from our sample with an Asian origin.

## Discussion

There has been limited research on the role of ROHs in autism in Asian populations. A recent study identified several novel candidate genes in ROH regions associated with the risk of autism in a European-descent population [15]. However, most of these loci reported by this study would not remain to be significantly associated with the disease risk after multi-testing corrections. Implementing stricter correction methods, we failed to detect significant disease-associated ROHs at a genome-wide level in our population. We speculate that the effect size of single ROH region associated with the risk of autism might be too small to be detected in a genome-wide scan. Another recent study reported that the length and number of ROHs in autistic cases were higher than controls in a southern European-descent population [26]. However, our study shows that either lengths of ROHs or Froh values were similar in cases and controls. The inconsistent findings may stem from the difference in the population history of different samples. Additionally, consanguinity is unlikely to explain the relationship between ROHs and speech delay, as our findings do not reveal a remarkable difference in the degree of inbreeding between subgroups with speech delay and without speech delay. Furthermore, recent positive selection may play a limited role in the ROHs associated with speech delay in autism, as none of the candidate genes were found to have a strong signature of selection sweeps. However, we found that the patterns of extended homozygosity decay from the core haplotype on 11q22.3 might vary by the presence of speech delay. Our results suggest the variant within the RAB39 gene might be associated with the variant within the CWF19L2 gene under recent positive selection.

The current findings reveal a few novel candidate genes on 11q22.3 associated with speech delay in a Taiwanese Han population of autism. Among these genes, NPAT and ATM genes are associated with ataxia telangiectasia, one of the most frequent autosomal recessive cerebellar ataxias. Ataxia telangiectasia is also characterized by impairment in verbal fluency. Individuals affected with Ataxia telangiectasia often show weak oral motor performance [27]. It is unclear whether ataxia telangiectasia and autism has similar defects in the speech pathologies. Another gene located in the same region, EXPH5 (exophilin 5) is a cerebellum-expressed gene [28]. Cerebellum modulates motor coordination that also regulates the speech function. Additionally, individuals with autism and speech delay and individuals with autism without speech delay have marked difference in metabolic ratio in cerebellar regions [29]. Therefore, the ATM, NPAT, and EXPH5 genes may influence some neural correlates associated with the cerebellum. Variants in these three genes may thus influence the language function linked to the cerebellum in autism.

Additionally, the CUL5 (culin 5) gene in the region has been found to regulate cortical layering by modulating the neuronal migration process [30], [31]. The protein culin 5 encoded by the CUL5 gene plays a pivotal role if degradation of an intracellular signaling molecule, Disabled-1, which is activated by reelin encoded by the RELN gene. Previous studies have shown mixed evidence for the association between the RELN gene and the risk of autism [32], [33]. It has been shown that subtle dysregulated neuronal migration, such as perisylvian polymicrogyria, is associated with the developmental language disorder [34]. An animal study also showed that homozygous mutants for the CUL5 variant is defective in Notch signaling as indicated by the impaired expression of Notch target genes, which affects the initiation of Notch signaling during neurogenesis [35]. These findings may comprise the lines of indirect evidence for the relationship between the CUL5 gene and speech delay.

The association of chromosome 11q structural variants with language impairment has been documented by several studies. For instance, at least half of the individuals afflicted by 11q terminal deletion syndrome might be affected by mild to moderate impairment in expressive language [36]. A case report documents a girl with a 11q21–22.3 deletion manifested multiple congenital abnormalities, including speech delay [37]. Two case studies also report the association between 11q24 deletion and developmental speech delay in Jacobson syndrome [38]. Mosaic 11q deletions have also been noted in metopic synostosis associated with an increased risk of speech delay [39]. Additionally, the deletion of 11q23.3 might be associated with speech delay [40], [41], while the duplication of the 11q23.3 region might also lead to speech delay [42]. Taken together, these findings suggest that the chromosome 11q21–q24 might harbor genes that play a role in language development.

Speech delay has been regarded as an endophenotype of autism. Some prior studies used speech delay as a clinical marker to identify homogeneous subgroups of autism, while others treated speech delay as an independent trait. Several regions have been found to be associated with speech delay in autism. For instance, the chromosome 7q31–q33 is one of the regions that have been found to contain genetic polymorphisms linked to speech delay in autism [43]–[47]. It has also been suggested that the 7q11–q12 duplication may be linked to speech delay in autism [48], [49]. Additionally, the chromosome 2q is another region that might contain genetic variants associated with speech delay in autism [50], [51]. Some of these candidate regions associated with speech delay in sporadic case reports. However, the CNTNAP2 (contactin associated protein-like 2) gene on 7q, which has been found to be linked to language impairment in some large-scale studies [52], [53], was not included in the ROH regions that were significantly associated with speech delay in our sample. The CNTNAP2 gene, as well as other candidate risk genes for autism, might not be identified in a case-only analysis of our study. It remains unclear if the molecular mechanisms of language impairment in individuals without autism differ from those in individuals with autism.

The current study has several limitations. First, the current study might not have sufficient power to detect variants of small to moderate effect on traits. This might at least partly explain the failure of our case-control association tests to replicate findings of previous studies. However, based on the parameters estimated in our case-control study, we achieved the statistical power of 30% given the α value = 0.0001. Second, the psychosocial factors that may influence language acquisition, such as parenting style and previous intervention, are not available in our samples. However, we did adjust for education levels of parents in the analysis and did not detect remarkable impact of parental education level on the genetic effect on clinical features. Nevertheless, parental education level might not fully reflect the quality of parenting and preschool education that may influence language acquisition. Third, the ROH based on the Affymetrix SNP 6.0 data might not consist of entirely homozygous SNPs, unless we have whole-genome sequencing data to verify these findings. Therefore, such a limitation might lead to the concern about the interpretation of our findings. Additionally, since we could only perform the analysis of clinical features in cases, our findings might not be generalized to the genetic basis for speech delay. However, our study has yielded some insight into the molecular basis for clinical heterogeneity in autism.

In contrast to the continuous outcome, the analysis based on the dichotomous outcome yielded relatively less significant results for the same region on 11q22.3. This mild inconsistency might imply that the variant on 11q associated with speech delay might lead to a more extremely speech delay. Therefore, the comparison between relatively extremely late age-of-first-phrase group and extremely early age-of-first-phrase group might yield a more remarkable difference in ROH distributions between the two subgroups. However, in the replication study, we noticed that the dichotomous outcome yielded a slightly stronger association signal than the continuous outcome. These findings suggest that more research is needed to investigate how to define “speech delay” based on the age of first phrase and genomic data.

To sum up, the current study suggests that novel candidate genes may yield a greater impact on speech delay compared to autism per se. Untangling the mechanisms of speech delay may shed some light on molecular mechanisms underlying the development of autism. The extended homozygous haplotypes associated with speech delay may be more likely to be attributable to the recessive disease model than selection sweeps or consanguinity in our sample. Our findings also suggest that susceptibility genes may not necessarily contribute to clinical heterogeneity in autism. Taken together, these findings may lead to the evidence-based classification algorithm for clinical subgroups. Finally, our findings suggest that a few cerebellum-associated genes may play a role in speech delay in autism. Multiple adjacent loci of these genes may act in concert to cause speech delay in autism. More research is warranted to investigate if any cerebellum-related pathological changes could predispose to speech delay in autism.

## Methods and Materials

### Ethics Statement

The protocol entitled “Clinical and molecular genetic studies of autism spectrum disorder”, submitted by Principle Investigator Dr. Susan Shur-Fen Gau, Department of Psychiatry, National Taiwan University Hospital, Taiwan, has been approved by the 119th meeting of Research Ethics Committee of the National Taiwan University Hospital on September 26, 2006 (NTUH-REC ID: 9561709027) and the other two collaborating sites (Chang-Gung Memorial Hospital in Taoyuan, CGMH ID: 93–6244 and Taoyuan Mental Hospital in Taoyuan, TYMH ID: C20060905). The committees of the three research sites were organized and operated according to GCP and the applicable laws and regulations. The Research Ethics Committee of three research sites approved this study [ClinicalTrials.gov number, NCT00494754]. Written informed consent was obtained from majority of the probands if they were able to give their signature after reading the informed consent and all their parents after the purposes and procedures of the study were fully explained and confidentiality was ensured. All subjects were Han Chinese. The data-sharing plan has been approved by all key investigators (SSG, YYW, and SKL) across three collaborating sites and approved by the Research Ethics Committee of the three sites. SSG, the principal investigator of this project, coordinated the research and managed all the clinical and genetic data. We reached the agreement that the de-identified data and key clinical variables will be released to investigators upon the request with relevant institutional approval documents.

#### Subject recruitment

The cases were selected from a sample of totally 1,164 subjects from 393 families (probands aged 9.1±3.99 years, male 88.6%), recruited from the outpatient clinic of Psychiatric Department of three institutes (i.e., National Taiwan University Hospital in Taipei, Chang-Gung Memorial Hospital in Taoyuan, and Taoyuan Mental Hospital in Taoyuan) in Northern Taiwan. Probands diagnosed with fragile X and Rett’s disorder based on DNA testing or clinical features were excluded (unpublished data). Additionally, probands with previously identified chromosomal structural abnormality associated with autism, or had any other major neurological or medical conditions were also excluded. The initial diagnoses of probands were made by senior board-certified child psychiatrists based on the DSM-IV diagnostic criteria of autistic disorder or Asperger’s disorder, and were further confirmed by interviewing the parents using the Chinese version of the Autism Diagnostic Interview-Revised (ADI-R) [54], adapted from the ADI-R [24]. The algorithm focuses on three domains based on the ICD-10 and DSM-IV diagnostic criteria, including reciprocal social interaction, verbal and non-verbal communication, as well as restricted, repetitive and stereotyped patterns of behaviors. We retrieved age of first phrase (AFP) to infer the presence of speech delay from the ADI-R assessment. AFP was treated as a continuous variable in the linear regression model that also controlled gender, SCQ, and parental education level. Additionally, we used k-means clustering algorithm with Euclidean distance to classify the sample into two subgroups, which were denoted as early-AFP group and late-AFP group.

The recruitment of controls was documented in detail elsewhere [55]. Briefly, the Institute of Biomedical Sciences, Academia Sinica and National Research Program for Genomic Medicine in Taiwan initiated the efforts to collect data to establish Han Chinese Cell and Genome Bank in Taiwan during 2002–2004. A three-stage sampling was implemented and complete bio-specimen and questionnaire data (with a focus on ethnicity and medical history) were collected for 3,380 individuals (gender ratio, 1∶1; age range, 20–70 years). A total of 1,115 individuals with a Han Chinese ancestry that were found to have no definite diagnosis of major medical or mental illnesses were treated as the controls for the current study.

#### Genotyping

All cases and controls were genotyped on Affymetrix SNP array 6.0 platform that could generate a maximum of 906,600 SNPs and 946,000 probes for the detection of CNVs (Affymetrix Inc., Santa Clara, CA, USA). The DNA samples were extracted and purified from the peripheral lymphocytes according to the manufacture’s protocol. Genotype calls for SNPs were made based on the Birdseed algorithm that performs a multi-chip analysis to estimate a signal intensity for each allele of each SNP [56]. The average call rate was 99.86%. We also performed the Hardy-Weinberg Equilibrium (HWE) test, and excluded the SNPs with a HWE *P*<5×10^−5^, so that the analysis would be less likely to be affected from genotyping or calling errors. A total of 546,080 SNPs were thereby analyzed in the association tests.

#### Association analysis

We defined an ROH as a stretch of DNA spanning at least 500 kb or 50 consecutive SNPs without any heterozygous SNPs. Additionally, the maximum gap between SNPs could not exceed 100 kb. The overlapped region of multiple ROH regions shared by at least 10 individuals was regarded as a core ROH region. Furthermore, the prevalence rate of each common ROH marker should be at least 1% in the controls. We performed a case-control analysis based on cases (n = 315) and controls (n = 1,115) to identify risk ROHs. We also compared the difference in the length of ROHs of case and controls by t-test. To further clarify the role of ROHs in the heterogeneity of language developmental function in autism, we also assessed the associations between ROHs and the AFP. The continuous outcome variable was regressed against each ROH marker using the linear regression model. To adjust for the impacts of parenting and other confounders, we controlled for educational levels of parents, performance IQ, and gender in each linear regression model. To alleviate the problem of over-fitting due to intra-collinearity, we also performed step-wise regression analysis for the most significant trait-associated ROH marker. To determine the significance level, we took into account the number of ROH markers and outcome variables and applied the conservative Bonferroni method to correct inflated type-I errors due to multiple tests (corrected genome-wide significance threshold = 0.05/N, N is the total number of OH regions). The ROH identification and association tests were performed using the software Golden Helix™ SNP and Variation Suite 7.6 (Golden Helix, Inc., Bozeman, MT, www.goldenhelix.com). Furthermore, we calculated the inbreeding coefficient F for sub-populations to assess if any spurious association arose from the difference in relatedness. Finally, we assessed if the size of gene might exert any impact on the association between trait-associated ROHs and traits by incorporating the gene size as a covariate in the regression model for the most significant finding.

#### Selection sweep analysis

The phase of the haplotypes in the trait-associated ROH region were constructed using the program of PHASE v 2.1 [57]. We limited the search of core haplotypes to the brain-expressed genes. We then calculated extended EHH (i.e., the probability that two randomly chosen chromosomes carrying the core haplotype of interest are identical by descent) to evaluate the evidence for selection sweeps. We further calculated the relative EHH value (REHH = core haplotype EHH divided by the decay of EHH on all other core haplotypes combined) to detect the signature of recent positive selection. We defined the evidence for selection sweep as REHH values ≥2 with long-range markers, radiating to distances greater than 200 kb from the core site, according to previous simulated data sets [58]. The phylogenic relationship among all possible core haplotypes was inferred by ancestral alleles. All of the analyses were performed using the software Sweep [58].

## Supporting Information

Figure S1 **The distributions of age of first phrase (AFP) of the discovery population (Taiwan) and replication population (AGRE) are shown.**
(TIFF)
