# Supplementary figures and images for: Runs of Homozygosity Associated with Speech Delay in Autism in a Taiwanese Han Population: Evidence for the Recessive Model
Source: PLoS One. 2013 Aug 16;8(8):e72056. doi: 10.1371/journal.pone.0072056 (PMC3745408; doi:10.1371/journal.pone.0072056)

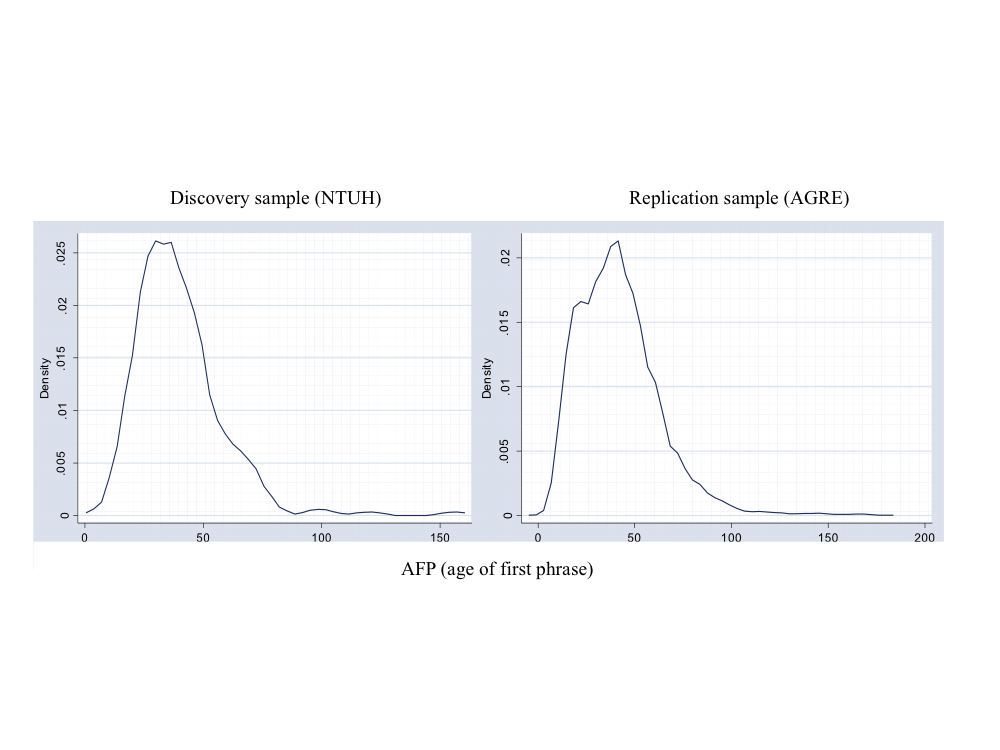

Supplement: Figure S1 — The distributions of age of first phrase (AFP) of the discovery population (Taiwan) and replication population (AGRE) are shown. (TIFF) [file pone.0072056.s001.tiff]
